# The fight between medicine and scepticism needs to be resolved by evidence: Book reviews

**DOI:** 10.1016/j.jsampl.2023.100042

**Published:** 2023-10-31

**Authors:** John Orchard

**Affiliations:** School of Public Health, University of Sydney, Australia

**Keywords:** Back pain, Evidence, Reimbursement, Exercise, Scepticism, Book reviews

## Abstract

Medical scepticism is on the rise worldwide. It is very important to differentiate between uneducated scepticism (e.g. the anti-vaccination movement) where valuable areas of medicine are disputed and well-informed scepticism, where legitimate experts reveal flaws in mainstream medicine. It is assumed that medicine being a science will be self-correcting and inevitably move towards a stronger evidence-base, but a competing factor is the profit motive. Three excellent books are reviewed, all Australian and all covering primarily musculoskeletal medicine (*Back Up* by Mannix; *Surgery: The Ultimate Placebo* and *Hippocrasy* by Harris and Buchbinder). These texts reveal that musculoskeletal medicine has many treatments where the desire to offer hope and, also, make a profit is taking precedence over scientific evidence. Because many healthcare presentations of young people are musculoskeletal, doctors risk losing the younger generation to medical scepticism in general if they continue to offer and promote flawed treatment options over effective ones. The most effective long-term treatments in musculoskeletal medicine (exercise load management and psychological reassurance) are less profitable because they require so much time investment. Health care professionals need to be true to their science background and aim to do only what is effective, not be drawn to drugs and procedures that are more profitable but ultimately more harmful.

## 1 Introduction

How much should medicine engage with scepticism? There is no simple answer. If you asked me to review a book that was sceptical about childhood vaccination, I'd refuse (and perhaps not even politely). Childhood vaccination is probably medicine's all-time greatest contribution to the health of the population [1]. Tremendous harm is done when either traditional media or social media gives “equal airtime” to anti-vaxxers, and reluctantly they are best dealt with by non-engagement. One of Upton Sinclair's quotes is “It is foolish to be convinced without evidence, but equally foolish to fail to be convinced by real evidence”. The evidence (some of it circumstantial but altogether compelling) is that childhood vaccination saves literally millions of lives for every child harmed [1]. In medicine you don't have to venture very far to find other huge wins: obstetric care, perinatal care, most of cardiology, most of endocrinology, much of oncology, infectious disease etc. If you work in these areas, you might have very little time for medical scepticism and the opinions of non-doctors who think they know better.

What if there were medical areas where most dogma was actually more harmful than beneficial? If you don't believe that such an area could exist, you need to read *Back Up: Why back pain treatments aren't working and the new science offering hope* by Liam Mannix [2]. At Australian newspapers *The Age* and *SMH,* Liam Mannix already has a reputation as a terrific journalist with huge respect for both science and medicine, yet his own and his father's experience with back pain has allowed him to write a book of intelligent scepticism. He intends to reform rather than destroy medicine, in an area where the current scoreboard is dismal. Mannix paints doctors as misguided when it comes to back pain rather than malevolent. Chiropractors tend to be derided by doctors on the basis that their profession was formed via magical thinking rather than science, yet it would be hard to read *Back Up* and not conclude that you'd be better off seeing a chiropractor and receive subluxations and reassurance than you would be to see a doctor and receive either a spinal fusion or opiate painkillers (and often they go hand in hand).

Australian doctor-authors Ian Harris (an orthopaedic surgeon) and Rachelle Buchbinder (a rheumatologist) have also recently written books (*Surgery: The Ultimate Placebo* [3] and *Hippocrasy: How doctors are betraying their oath* [4]), through the same publisher as Mannix. They also mount the case that across musculoskeletal medicine there are perhaps more losses (harmful or placebo treatments) than there are actual wins (beneficial treatments like fracture fixation for displaced limb fractures, and disease-modifying treatment for gout and rheumatoid arthritis) [4]. In these great books they explore the paradox that the *majority of surgical patients get better*; however, *at least as many placebo surgery cases get better*. Clearly something is going on that isn't the actual surgical technique. Is it pure placebo (the expectation that the ‘best’ treatment will get the result)? Or is it mainly contextual effects of surgery, say, that rehab plans are strictly adhered to when post-surgery but poorly adhered to without surgery? The title of *Hippocrasy* makes it clear that Buchbinder and Harris think that individual doctors bear the lion's share of the blame for why ineffective surgeries (and drugs) are still getting used. A highlighted procedure is knee arthroscopy which is clearly either a placebo procedure (on a good day) or a harmful one (on a bad day) [5]. Knee pain treatments which are harmful (knee arthroscopy and opiates) are generously funded in Australia, and most countries [6], whereas advice on exercise, amongst the most beneficial of treatments for musculoskeletal conditions, is poorly funded or not funded [7]. Doctors are more likely to prescribe ineffective/harmful drugs and procedures than give advice on exercise [7]. Upton Sinclair's even more well-known quote is relevant here: “that it is difficult to get someone to understand something if their salary depends on them not understanding it”. Buchbinder and Harris use an appropriate neologism “Hippocrasy” (betraying the doctor's Hippocratic Oath to “do no harm”). All three books touch on the topic of how doctors are complicit in corrupting the reimbursement system in medicine to make sure that bad treatments continue to get funded. However, the vilification of whistleblowers who have publicly claimed that Medicare fraud in Australia is commonplace perhaps explains why it is such a difficult topic to approach.

There is not (yet) a book which covers the topic of why exercise (prescribed as “load management”) [8,9] – as a medical treatment – is denied much funding when we know it is the best primary treatment for musculoskeletal conditions (and it can also treat cardiovascular disease, diabetes, depression and even cancer) [10]. It is apparently “impossible” to stop funding spinal fusion and opiates, but in the Australian sports medicine world we've been witness to cuts in rebates for long consultations for sport and exercise physicians [7]. The difficulty in funding exercise as a medical treatment is not really explored by Mannix [2]. He does note, fairly, that in funding exercise treatment via physiotherapy you may be funding ineffective dogma (posture control, fixing one's “core” or ergonomics) as much as advice on the psychological aspects of back pain [11] or the right dosing of exercise (the effective treatments).

From a Sport and Exercise medicine (SEM) perspective, it appears that we have neither enough overlap with mainstream medicine nor the world of back pain research. It is either likely or possible that SEM/sports physiotherapy has found one of the few areas in back pain (lumbar stress fractures in teenage athletes) where structural management is effective. In the 1980s, over 80 ​% of Australian fast bowlers had unhealed stress fractures of the lumbar spine, whereas in the modern era, over 80 ​% of elite fast bowlers who get stress fractures manage to have them heal [12,13]. We (in SEM) assume that this makes them better bowlers with longer careers, but we only have the cohort evidence - not RCT evidence - to argue the case. The topic of lumbar stress fractures – which barely rates a mention in Mannix's book - challenges SEM and back pain researchers to collaborate more in the future.

The three texts are all excellent reading, as are some other recent books in the medical scepticism genre [[14], [15], [16]]. They should lead to more discussion about why there is excess scepticism about areas of medicine that don't warrant it (childhood vaccination, water fluoridation, COVID prevention) and not enough scepticism within areas of medicine that are crying out for it (musculoskeletal medicine). Perhaps when parts of medicine prioritise profit over patient outcomes (e.g. opiates for non-cancer pain; ineffective surgeries) it can breed reactionary scepticism in the areas of medicine where traditional healthcare is lifesaving. Pfizer, for example, are the makers of Lyrica, a drug correctly characterised by Mannix as being all side effect and expense with no benefit [2,17]. By funding Lyrica (which health systems still do, despite its lack of any demonstrable benefit) we give any member of the public who's been burnt by this drug every reason to be sceptical of medicine/big Pharma, so when Pfizer's “good” (lifesaving) drugs are offered to them (Paxlovid and COVID vaccines) they may already have passed judgment that they want nothing to do with them.

Education of all of the public, mainstream media and healthcare professions needs to focus on evidence for benefits in the longer-term [18], not the immediate term. The great benefits of vaccination are delayed, whereas any side effects are immediate, and it takes trust in medicine to accept the trade-off (which is enormous). Exercise therapy offers a similar enormous, but often delayed, benefit, whereas opiates and other painkillers (including cortisone injections) [19] offer a short-term benefit with the harm delayed.

The medium-to-long-term harms are not trivial. Scepticism about COVID vaccines has been responsible for the deaths of thousands of Republicans in the USA [20]. But equally, trust in doctors who have prescribed opiate painkillers has led to thousands of deaths in many countries of the world [21,22].

A fair conclusion is that medicine is full of both many miracles and quite a few warts. Healthy scepticism is certainly required if you are a health practitioner, but so is optimism/compassion. I applaud Harris and Buchbinder for their bravery in writing texts from the position of being doctors exposing their own profession. Liam Mannix with *Back Up* [2] takes the baton from the two doctors and runs a bit further with a very readable exploration of a single important topic (back pain). The genre of medical scepticism has even further to still run, with the end aim being a health system where evidence-based treatments are funded and ineffective treatments are discarded. Sadly in musculoskeletal medicine we can't say we are there yet, and fighting against the profit motive will mean it is a long battle.

## Ethics considerations

This is a book review submission, so no requirement for Ethics Committee oversight.

## Funding

There is no funding to declare for these book reviews. The author purchased all books to read and was not provided with free copies for the purposes of review.

## Declaration of competing interest

There are no conflicts between the author, JWO, and any of the material covered in the books under review.
